# Two *Blautia* Species Associated with Visceral Fat Accumulation: A One-Year Longitudinal Study

**DOI:** 10.3390/biology11020318

**Published:** 2022-02-16

**Authors:** Naoki Ozato, Tohru Yamaguchi, Kenta Mori, Mitsuhiro Katashima, Mika Kumagai, Koichi Murashita, Yoshihisa Katsuragi, Yoshinori Tamada, Masanori Kakuta, Seiya Imoto, Kazushige Ihara, Shigeyuki Nakaji

**Affiliations:** 1 Department of Active Life Promotion Sciences, Graduate School of Medicine, Hirosaki University, Hirosaki City 036-8562, Japan; mori.kenta@kao.com (K.M.); katashima.mitsuhiro@kao.com (M.K.); kumaga20@osaka-cu.ac.jp (M.K.); katsuragi.yoshihisa@kao.com (Y.K.); 2 Health & Wellness Products Research Laboratories, Kao Corporation, Tokyo 131-8501, Japan; yamaguchi.tohru@kao.com; 3 COI Research Initiatives Organization, Graduate School of Medicine, Hirosaki University, Hirosaki City 036-8562, Japan; murasita@hirosaki-u.ac.jp; 4 Innovation Center for Health Promotion, Hirosaki University, Hirosaki City 036-8562, Japan; y.tamada@hirosaki-u.ac.jp; 5 Human Genome Center, Institute of Medical Science, University of Tokyo, Tokyo 113-8654, Japan; mkakuta.dsc@tmd.ac.jp (M.K.); imoto@hgc.jp (S.I.); 6 M&D Data Science Center, Tokyo Medical and Dental University, Tokyo 113-8510, Japan; 7 Department of Social Medicine, Graduate School of Medicine, Hirosaki University, Hirosaki City 036-8562, Japan; ihara@hirosaki-u.ac.jp (K.I.); nakaji@hirosaki-u.ac.jp (S.N.)

**Keywords:** visceral fat, body mass index, intestinal microflora, obesity, cardiovascular disorder, *Blautia* *hansenii*, *Blautia* *producta*

## Abstract

**Simple Summary:**

Intestinal microflora has been associated with obesity. While cardiovascular disorders are more strongly associated with visceral fat than the body mass index (BMI), the link between visceral fat area (VFA) and intestinal microflora has been little studied. In this study, we investigated the association between intestinal microflora and VFA and BMI using a longitudinal study (N = 767). We found that the intestinal microflora composition is significantly associated with VFA or BMI; however, the associated gut microbes differ. Furthermore, two gut species—*Blautia hansenii* and *Blautia producta*—were significantly and negatively associated with VFA accumulation.

**Abstract:**

Intestinal microflora has been associated with obesity. While visceral fat is more strongly associated with cardiovascular disorder, a complication linked to obesity, than the body mass index (BMI), the association between intestinal microflora and obesity (as defined in terms of BMI) has been studied widely. However, the link between visceral fat area (VFA) and intestinal microflora has been little studied. In this study, we investigate the association between intestinal microflora and VFA and BMI using a longitudinal study on Japanese subjects with different VFA statuses (*N* = 767). Principal component analysis of the changes in intestinal microflora composition over the one-year study period revealed the different associations between intestinal microflora and VFA and BMI. As determined by 16S rRNA amplicon sequencing, changes in the abundance ratio of two microbial genera—*Blautia* and *Flavonifractor*—were significantly associated with VFA changes and changes in the abundance ratio of four different microbial genera were significantly associated with BMI changes, suggesting that the associated intestinal microbes are different. Furthermore, as determined by metagenomic shotgun sequences, changes in the abundance ratios of two *Blautia* species—*Blautia* *hansenii* and *Blautia* *producta*—were significantly and negatively associated with VFA changes. Our findings might be used to develop a new treatment for visceral fat.

## 1. Introduction

The intestinal microflora resides in one of the largest interfaces between the host, environmental factors, and antigens in the human body. It is strongly associated with host energy regulation and homeostasis, contributing to obesity and diabetes status [1]. According to some studies, intestinal microflora is a contributing factor to the development of obesity [2,3,4,5]. Furthermore, intestinal microflora depends on environmental factors, such as diet [6,7,8], age [9,10], and gender [11,12].

As revealed by studies involving animals, intestinal microflora impacts insulin resistance and adiposity [13,14]. In humans, four major phyla—Bacteroidetes, Firmicutes, Actinobacteria, and Proteobacteria—account for approximately 98% of the intestinal microflora. In some studies, subjects classified as obese based on their body mass index (BMI), were shown to harbor significantly fewer Bacteroidetes than non-obese subjects [15,16]; however, other studies reported conflicting findings [17,18,19,20]. Based on a previous study of more than 1000 subjects, we proposed that gender differences might be one of the reasons for this inconsistency [21]. Others have suggested the importance of the gut microbiome composition [15,22,23] and lack of microbial diversity [22,24]. However, almost all of these were cross-sectional studies or studies involving only female subjects. Furthermore, BMI has been widely used as a proxy for obesity in studies focusing on intestinal microflora and obesity.

Visceral fat accumulation is well-known as a risk factor for cardiovascular disorder [25,26] and all-cause mortality [27,28,29], independent of BMI and general obesity. Furthermore, visceral fat area (VFA) is associated more strongly with metabolic risk factors, such as hypertension, high blood concentrations of glucose and triglyceride, and low serum high-density lipoprotein cholesterol, than BMI [30,31]. Therefore, VFA accumulation could increase the risk of metabolic syndrome-related disease. Hence, compared with the association between intestinal microflora and BMI, the association between intestinal microflora and VFA might be more clinically important.

In this study, a one-year longitudinal study was performed involving 767 Japanese subjects. We investigated the association between intestinal microflora and VFA using a bio-impedance-type visceral fat meter [32], as well as BMI. We show that the intestinal microflora differently impacts VFA and BMI, and that specific microbial species may potentially be used to improve individual VFA status.

## 2. Methods

### 2.1. Design, Study Subjects and Ethics

**Figure 1:**
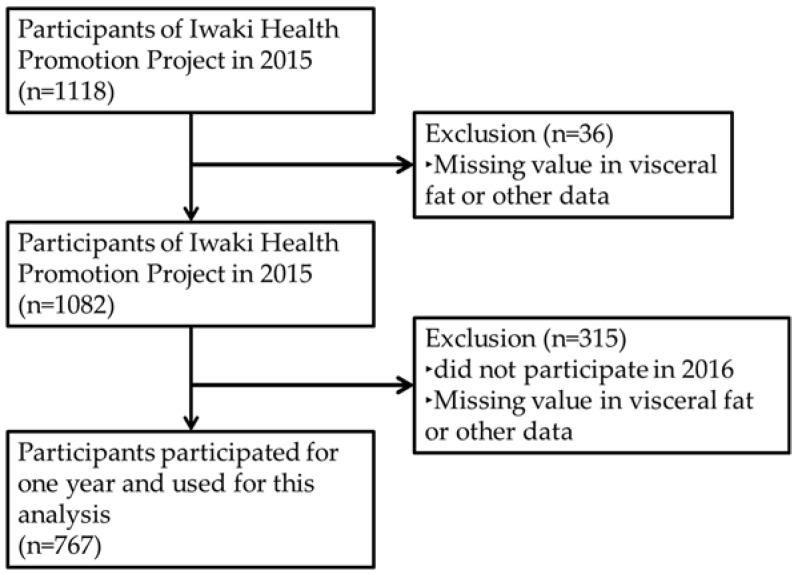
Study flow of the subjects. A total of 767 subjects completed clinical assessments for one year and were enrolled in this study.

In 2005, the Iwaki Health Promotion Project was launched as an annual health check-up program. Subjects were adult men and women living in the Iwaki region of Hirosaki City in Aomori Prefecture, Japan [21,33,34,35,36,37,38]. In 2015, VFA was measured for the first time and this study was designed as a population-based longitudinal study, using data obtained from May 2015 and May 2016. For baseline study, 1118 individuals took part in this health check-up (Figure 1). Of these, 36 subjects did not complete the clinical assessment, such as intestinal microflora data, and/or VFA measurements, and were excluded from subsequent analysis. Furthermore, 315 subjects with missing intestinal microflora data and/or VFA data for 2016 were excluded. Ultimately, 767 subjects (311 males and 456 females) were enrolled in the analysis.

### 2.2. Fecal Sample and DNA Extraction

Two to three grams of fecal samples were collected by each subject using an FS-0002 tube kit (TechnoSuruga Laboratory Co. Ltd., Shizuoka, Japan) containing a stock guanidine thiocyanate solution (100 mM Tris–HCl (pH 9.0), 40 mM Tris–EDTA (pH 8.0), and 4 M guanidine thiocyanate) at both annual health check-ups (2015 and 2016) within 3 d prior to the study. After collecting the sample, we asked the subject to store it in a refrigerator at 4 °C or less until the annual health check-up.

Then, 800 μL GTC buffer solutions containing fecal samples were transferred to 2 mL tubes filled with zirconium beads. Fecal sample suspensions were milled with zirconia beads at 5 m/s for 2 min using the FastPrep 24 Instrument (MP Biomedicals, Santa Ana, CA, USA). The samples were then cooled and centrifuged for 1 min at 2350× *g*. DNA was extracted from 200 μL aliquots of the samples using the automated Magtration System 12Gc with MagdDEA DNA 200 as the reagent.

### 2.3. Polymerase Chain Reaction (PCR) and Sequencing for 16S rRNA Gene Sequences

The sequence of the V3–V4 region of 16S rDNA was used to identify bacteria and the PCR mixture and conditions were maintained as described previously [39]. Then, the PCR mixtures were resolved on 1.0% agarose gels to separate the fragments, and PCR Cleanup Filter Plates (Merck Millipore, Burlington, MA, USA) were used to purify the fragments. The concentration of the PCR fragments was determined using real-time quantitative PCR, as described previously [39]. The MiSeq system (Illumina, San Diego, CA, USA) and 2 × 300 cycle paired-end method were used for Illumina paired-end sequencing.

### 2.4. Taxonomic Classification of 16S rRNA Gene Sequences

The amplified paired-end reads obtained by sequencing were processed, as described previously [21]. In brief, Cutadapt (version 1.13) was used to trim the adapter sequences and low-quality bases (threshold = 20) at the 3′ read-end, and the reads shorter than 150 bases and those containing N bases were discarded. Paired-end reads above the filter threshold were merged to form a single read (the “merged read”), and the merged reads longer than 470 bases or shorter than 370 bases were excluded by the fastq_mergepairs sub-command in VSEARCH (version 2.4.3). Furthermore, we excluded the merged reads with more than one sequencing error. The merged reads that remained after removing the chimera reads detected by the uchime_denovo sub-command in VSEARCH were clustered at a sequence identity greater than or equal to 97%. Taxa in the identified clusters were determined by applying the RDP Classifier to analyze the representative reads. Results with a confidence value under 0.8 were treated as unclassified.

### 2.5. Polymerase Chain Reaction (PCR) and Sequencing for Metagenomic Shotgun Sequences

The quality and quantification assessment of extracted DNA derived from fecal samples were measured by a 2200 TapeStation System (Agilent Technologies, Santa Clara, CA, USA). DNA samples collected from all subjects were then subjected to library preparation using the TruSeq ChIP Library Preparation Kit (Illumina). In brief, DNA samples were fragmented using an LE220 (Covaris, MA, USA), end-repaired, 3′-A added, ligated with adaptors, and amplified by PCR. The size distribution of the resultant libraries was measured using a 2200 TapeStation System and quantified by quantitative PCR using KAPA Library Quantification Kits (KAPA Biosystems, Wilmington, MA, USA). Sequencing was performed on a HiSeq2500 instrument (Illumina) with 101 bp paired-end mode.

### 2.6. Taxonomic Classification of Metagenomic Shotgun Sequences

The adapter sequences and low-quality 3′-terminal regions were trimmed from paired-end reads by Cutadapt (version 1.13) with a quality threshold of 30. Reads shorter than 80 bases and reads from the host genome (GRCh38) were discarded. We considered both reads that were mapped to the host genome with 80 match lengths or more by BWA-MEM (version 0.7.15) to be from the host genome. The remaining reads were taxonomically classified by Centrifuge (version 1.0.4-beta).

### 2.7. Other Measurements

All of the study subjects underwent a health check-up following at least 9 h of fasting. A bio-impedance-type visceral fat meter, EW-FA90 (Panasonic Corporation, Osaka, Japan), authorized as a noninvasive medical device (number 22500BZX00522000) was used to measure VFA. The measurements obtained using this visceral fat meter were comparable to those obtained using computed tomography (R > 0.8) [32]. Moreover, the following clinical characteristics were evaluated: body weight, height, BMI (calculated from height and body weight), waist circumference, diastolic blood pressure (DBP), systolic blood pressure (SBP), total serum cholesterol concentration, TG, and high-density lipoprotein (HDL) cholesterol, low-density lipoprotein (LDL) cholesterol fasting serum glucose, HbA1c. All the laboratory tests were carried out by LSI Medience Co. (Tokyo, Japan) and conducted according to their standard operating procedure. Blood samples were collected from the peripheral vein. Smoking amounts (cigarettes/d) and habitual medicine use (e.g., medicine for diabetes, hyperlipidemia, hypertension, rheumatism, dementia, or allergies) were obtained using self-administered questionnaires or daily journals. Furthermore, the daily intake of carbohydrate, protein, fat, alcohol, and total dietary fiber was calculated using the Brief Diet History Questionnaire [40,41].

### 2.8. Statistical Analysis

In this study, subject characteristics are represented as a percentage or mean ± standard deviation (SD). The Mann–Whitney U-test was used to compare two subject groups, whereas the exact Jonckheere test was used for more than two subject groups. Shannon diversity is one of the most commonly reported diversity metrics and weights the numbers of species by their relative evenness data. Therefore, to show the changes in intestinal microflora composition the Shannon index was used. Principal component analysis is often used as a tool in exploratory data analysis for variable dimensionality reduction and can be used to reduce a high number of predictor variables to a few principal components. In our study, principal component 1 (PC1) to PC5 were considered [42,43]. The association between changes in intestinal microflora composition using scores of principal component analysis and changes in VFA or BMI was determined by analysis of variance for a linear regression model with VFA or BMI as an objective variable, and intestinal microflora and covariates (e.g., lifestyle habits, gender and age) as explanatory variables. To determine the genera or species associated with the changes in VFA or BMI, multiple regression analysis was used, with the change in VFA or BMI as an objective variable and intestinal microflora and covariates (e.g., age and lifestyle habits) as explanatory variables. Statistical tests were two-tailed, and results with *p* < 0.05 were considered significant. R software version 3.3.4 was used in all analyses.

## 3. Results

### 3.1. Baseline Characteristics of This Longitudinal Study

A total of 767 subjects (59.5% female) were enrolled in this longitudinal study. At baseline, 28.6% of males and 18.4% of females were overweight (defined as BMI ≥ 25). These overweight rates were comparable with those reported by the Japanese Government in 2010 (obesity and overweight rate was 33.5% for males and 20.5% for females). The mean VFA was 106.5 ± 43.2 cm^2^ in males and 67.7 ± 31.9 cm^2^ in females; it was higher in males and lower in females, compared to the value defined as visceral obesity (≥100 cm^2^) [44]. Based on the median VFA, the subjects were divided into two groups: a high-VFA group (225 males and 155 females) and a low-VFA group (86 males and 301 females; see Table 1). In regard to the metabolic risk factors, the high-VFA group was significantly higher for glucose (*p* < 0.001), glycated hemoglobin (HbA1c, *p* = 0.001), systolic blood pressure (SBP, *p* = 0.001), diastolic blood pressure (DBP, *p* < 0.001), TG (*p* < 0.001), and low-density lipoprotein (LDL) cholesterol (*p* < 0.001) levels than the low-VFA group. However, HDL cholesterol levels were significantly lower in the high-VFA group than in the low-VFA group (*p* = 0.001). As for dietary habits, the high-VFA group used significantly higher energy and alcohol than the low-VFA group (*p* = 0.002 and *p* < 0.001, respectively). The intake of total dietary fiber was not significantly associated with VFA.

### 3.2. Changes in Intestinal Microflora Composition, VFA and BMI over the One-Year Study Period

**Figure 2:**
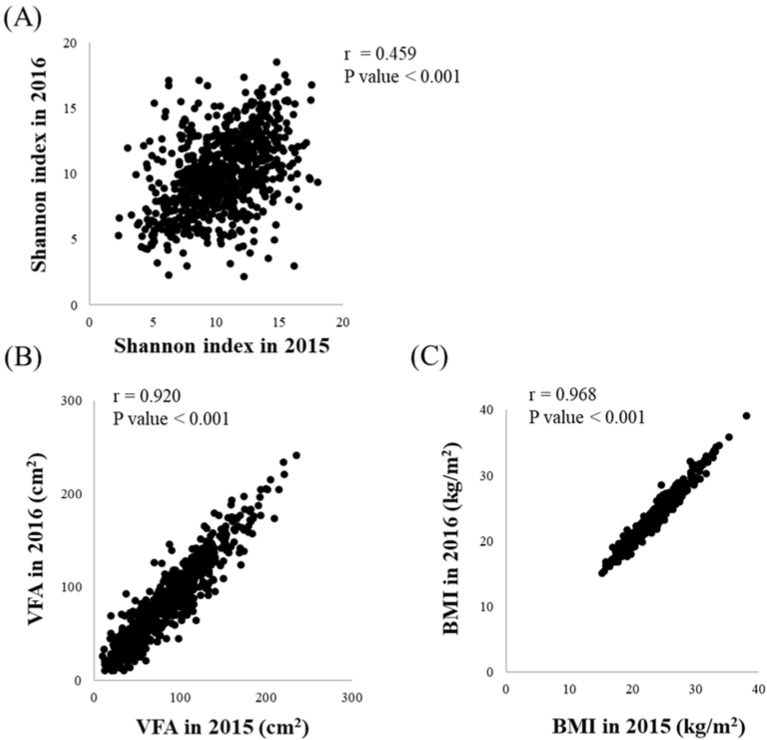
Changes in intestinal microflora, VFA, and BMI observed over one year (2015 to 2016, *N* = 767): (**A**) changes in intestinal microflora composition; (**B**) changes in VFA; (**C**) changes in BMI. Associations between variables were evaluated using Spearman correlation.

To investigate the changes in intestinal microflora composition, as well as changes in VFA and BMI, we compared the composition of gut microbes (Shannon index) and VFA and BMI values in 2015 with those in 2016 (Figure 2). The changes in intestinal microflora were assessed by 16S rRNA amplicon sequencing, while changes in VFA and BMI were determined using standard procedures. Subjects with a higher Shannon index in 2015 exhibited a significantly higher Shannon index in 2016, but the relationship was not strong (*r* = 0.459, *p* < 0.001). Four major phyla—Bacteroidetes, Firmicutes, Actinobacteria, and Proteobacteria—account for the majority (approximately 98%) of the human intestinal microflora. The abundance ratio of each phylum in 2015 and 2016 is shown in Figure S1. The abundance ratio of each phylum in 2015 and 2016 was significantly and positively associated, but the association was not strong (*r* = 0.515, *p* < 0.001 for Firmicutes; *r* = 0.482, *p* < 0.001 for Bacteroidetes; *r* = 0.602, *p* < 0.001 for Actinobacteria; *r* = 0.443, *p* < 0.001 for Proteobacteria).

In addition, the VFA values in 2015 and 2016 were significantly and positively associated with a strong relationship (*r* = 0.920, *p* < 0.001). We made similar observations for the BMI values in 2015 and 2016 (*r* = 0.968, *p* < 0.001). Furthermore, during the study period, the intestinal microflora composition varied to a greater extent than the VFA or BMI values.

### 3.3. Association between Changes in Intestinal Microflora Composition and Changes in VFA or BMI

**Figure 3:**
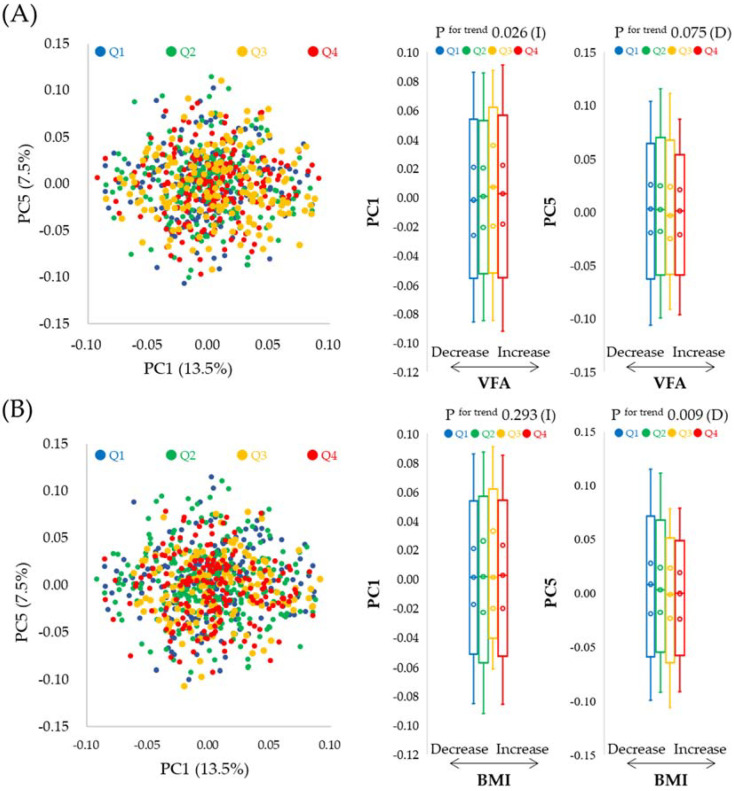
Association between changes in intestinal microflora composition and changes in VFA or BMI. Principal component analysis was performed; PC1 and PC5 data are shown, as PC1 and PC5 were significantly associated with VFA and BMI, respectively. Box plots are used to show these associations: (**A**) Changes in intestinal microflora composition at the genus level and changes in VFA. Subjects were divided into quantiles, based on changes in VFA: Q1 ≤ −10 (*N* = 203, blue); −10 < Q2 ≤ 0 (*N* = 207, green); 0 < Q3 ≤ 8 (*N* = 166, orange); 8 < Q4 (*N* = 191, red). (**B**) Changes in intestinal microflora composition at the genus level and changes in BMI. Subjects were divided into quantiles according to BMI: Q1 ≤ −0.2 (*N* = 194, blue); −0.2 < Q2 ≤ 0.3 (*N* = 251, green); 0.3 < Q3 ≤ 0.6 (*N* = 137, orange); 0.6 < Q4 (*N* = 185, red). The trend in *p* values was determined using the Jonckheere test. (I) indicates a tendency to increase, while (D) indicates a tendency to decrease in relation to VFA or BMI.

We observed that the intestinal microflora composition changed during the study period (Figure 2 and Figure S1). Next, we investigated the association between changes in intestinal microflora composition and changes in VFA or BMI. We analyzed the changes in the abundance ratio of detected gut microbial genera (305 genera) with principal component analysis using 16S rRNA amplicon sequencing. We divided the subjects into quantiles according to the changes in VFA or BMI. The association between each principal component score, from principal component 1 (PC1) to PC5, and the changes in VFA or BMI are shown in Table S1. The variance in changes in the intestinal microflora composition was explained by each PC; 13.5% for PC1, 11.8% for PC2, 9.4% for PC3, 8.2% for PC4 and 7.5% for PC5. The changes in VFA were significantly and positively associated with PC1; however, they were not associated with the other PCs. By contrast, changes in BMI were significantly and negatively associated with PC5 but were not associated with other PCs. Therefore, we selected PC1 and PC5 to probe the association between changes in the intestinal microflora composition and changes in VFA or BMI, respectively (Figure 3).

**Figure 4:**
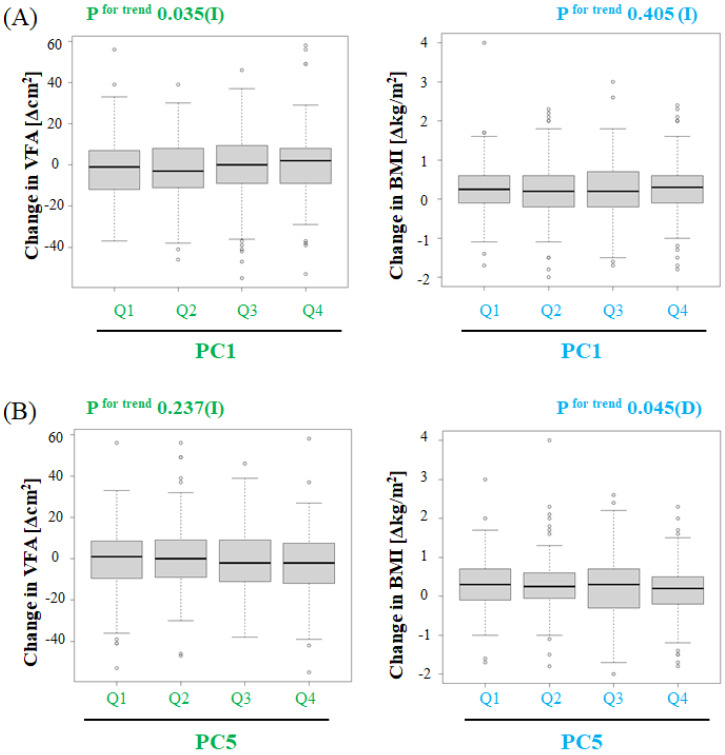
Effect of changes in intestinal microflora composition on VFA or BMI: (**A**) Effect of changes in intestinal microflora composition (PC1) on VFA or BMI. Subjects were divided into quantiles according to PC1 scores: Q1 ≤ −0.0212 (*N* = 191); −0.0212 < Q2 ≤ 0.00129 (*N* = 192); 0.00129 < Q3 ≤ 0.0253 (*N* = 192); 0.0253 < Q4 (*N* = 192). (**B**) Effect of changes in intestinal microflora composition (PC5) on VFA or BMI. Subjects were divided into quantiles based on PC5 scores: Q1 ≤ −0.0217 (*N* = 191); −0.0217 < Q2 ≤ 0.00188 (*N* = 192); 0.00188 < Q3 ≤ 0.0227 (*N* = 192); 0.0227 < Q4 (*N* = 192). For VFA, the trend in *p* values was determined by the analysis of variance for a linear regression model, where the change in VFA was the objective variable and age, gender, VFA, and Shannon index at baseline, as well as changes in alcohol consumption, total fiber intake, smoking amount, amount of exercise, and medicine use, were explanatory variables. For BMI, the trend in *p* values was determined by the analysis of variance for a linear regression model, where the change in BMI was the objective variable and age, gender, BMI, and Shannon index at baseline, as well as changes in alcohol consumption, total fiber intake, smoking amount, amount of exercise, and medicine use, were explanatory variables. (I) indicates a tendency to increase, while (D) indicates a tendency to decrease in relation to VFA or BMI.

To evaluate the association between the changes in intestinal microflora composition and changes in VFA or BMI, we divided the subjects into quantiles based on their PC1 or PC5 scores and verified the association of the changes in the intestinal microflora composition with changes in VFA or BMI. For VFA, we assessed the trend *p*-values by the analysis of variance of a linear regression model, with changes in VFA as an objective variable and age, gender, VFA, and Shannon index at baseline, as well as changes in alcohol consumption, total fiber intake, smoking amount, amount of exercise, and medicine use as explanatory variables. For BMI, we assessed the trend in *p*-values by the analysis of variance of a linear regression model, with changes in BMI as an objective variable and age, gender, BMI, and Shannon index at baseline, as well as changes in alcohol consumption, total fiber intake, smoking amount, amount of exercise, and medicine use as explanatory variables. PC1 was significantly and positively associated with changes in VFA (*p* = 0.035), but not with BMI, after adjustment for the above factors (Figure 4A). However, PC5 was significantly and inversely associated with changes in BMI (*p* = 0.045), but not with VFA, after adjustment for the above factors (Figure 4B). These observations suggest that both VFA and BMI are associated with the intestinal microflora at the genus level; however, the association between intestinal microflora and VFA or BMI was different.

### 3.4. Intestinal Microflora Genera Associated with VFA or BMI over the One-Year Study Period

We observed that the intestinal microflora composition associated differently with VFA and BMI. Therefore, we next investigated the specific gut microbial genera, assessed by 16S rRNA amplicon sequencing, which were significantly associated with VFA or BMI. Overall, we detected 305 genera in the analyzed samples; however, the relative abundance ratio of some genera was below 0.01% at the baseline, which was too small to detect accurately, so we excluded these genera from further analysis. Consequently, we included 36 genera in the analysis. The associations between changes in the abundance ratio of gut microbial genera and changes in VFA or BMI are shown in Table S2. However, there are many confounding factors such as age and gender in these associations. Therefore, the associations between changes in the abundance ratio of gut microbial genera and changes in VFA or BMI were adjusted by confounding factors (Table 2 and overall data are presented in Table S3). For VFA, the following factors were used as confounding factors: age, gender, VFA, and the abundance ratio of each genus at the baseline (model 1). For BMI, the following factors were used as confounding factors: age, gender, BMI, and the abundance ratio of each genus at baseline (model 2).

The changes in the abundance ratios of two genera were significantly associated with changes in VFA after adjusting for model 1. The changes in VFA were significantly and inversely associated with changes in the abundance ratios of *Blautia* and *Flavonifractor* (β = −36.2, *p* = 0.015; and β = −486.1, *p* = 0.016, respectively). The changes in the abundance ratios of four genera were significantly associated with changes in BMI after adjusting for model 2. The changes in BMI were significantly and inversely associated with changes in the abundance ratios of *Alistipes* (β = −4.17, *p* = 0.038), *Clostridium XlVb* (β = −30.69, *p* = 0.019), *Erysipelotrichaceae incertae sedis* (β = −46.21, *p* < 0.001), and *Lactobacillus* (β = −5.57, *p* = 0.014). This confirmed that specific gut microbial genera were significantly correlated with VFA and BMI values.

Next, to analyze the relationship between gut microbial genera, *Blautia* and *Flavonifractor*, and VFA in more detail, the following factors were adjusted in addition to model 1: changes in alcohol consumption, total fiber intake, smoking amount, exercise amount, BMI, and habitual medicine use, which are known to affect the intestinal microflora composition or VFA (model 3). After adjusting for model 3, the data were similar to those obtained after adjusting for model 1 (β = −34.7, *p* = 0.017 for *Blautia* and *β* = −435.4, *p* = 0.026 for *Flavonifractor*). Furthermore, multiple regression analysis of the association between the change in VFA and changes in *Blautia* or *Flavonifractor*, including related factors are shown in Table S4.

### 3.5. Gut Microbial Species Associated with VFA over the One-Year Study Period Using Metagenomic Shotgun Sequences

We used metagenomic shotgun sequences to investigate the species in *Blautia* and *Flavonifractor* significantly associated with VFA accumulation (Table 3). In our method, we detected five *Blautia* species—*Blautia producta*, *Blautia hansenii*, *Blautia* sp. *N6H1.15*, *Blautia* sp. *SC05B48*, and *Blautia* sp. *YL58*—and one *Flavonifractor* species, *Flavonifractor plautii*. To assess these species, the following factors were used for the adjustment: age, gender, VFA, the abundance ratio of each intestinal microflora species at baseline, changes in alcohol consumption, total fiber intake, smoking amount, exercise amount, BMI, and habitual medicine use. As for the *Blautia* genus, the changes in the abundance ratios of two species—*Blautia hansenii* and *Blautia producta*—were significantly and negatively associated with changes in VFA (β = −8.31, *p* = 0.001 and β = −26.16, *p* = < 0.001, respectively). However, the changes in the abundance ratios of the other three species were not significantly associated with changes in VFA. As for the *Flavonifractor* genus, the changes in the abundance ratio of *Flavonifractor plautii* were not significantly associated with changes in VFA (β = −1.28, *p* = 0.131).

## 4. Discussion

The current study is the first scientific longitudinal study focused on the association of VFA or BMI with intestinal microflora using a high number of subjects (767 subjects). We found that various intestinal microbial genera are differently associated with VFA and BMI and, in particular, found that two *Blautia* species were significantly and negatively associated with VFA accumulation.

The composition of the intestinal microflora is notably affected by ethnicity [45]. Our data on the abundance ratios of the four major phyla—namely, Bacteroidetes, Firmicutes, Actinobacteria, and Proteobacteria—in the gut were similar to those previously reported using a Japanese population [45]. However, in other populations, such as North American, Chinese, and Russian, the abundance ratio of Actinobacteria is lower than in the Japanese population and that of Proteobacteria is higher [46].

Intestinal microflora composition changed over the course of a year (Figure 2 and Figure S1). In the current study, we investigated the association between changes in intestinal microflora composition and changes in VFA or BMI. In our cross-sectional study, we found that the intestinal microflora composition was significantly associated with VFA or BMI; however, the associated gut microbes were different, even after adjusting for age, gender, alcohol consumption, and total fiber intake, which might affect the perceived associations (Figure 2 and Figure 3). Many studies involving humans have suggested that the intestinal microflora contributes to obesity status [1,15,17,18,19,20,21]; the findings of the current longitudinal study support these observations. VFA correlates with BMI but varies with race [47]. Hence, the current study suggests that VFA might be a confounding factor when assessing the relationship between BMI and specific gut microbes, or VFA and BMI, making it difficult to explain the inconsistencies between studies; however, further investigation is required in different populations including those of different race.

VFA is a major predictor of cardiovascular disorder [25,26] and is highly associated with metabolic risk factors [47], independent of BMI. Furthermore, the intestinal microflora is highly associated with host energy regulation and homeostasis, thereby contributing to obesity or diabetes status [1]. The difference in specific gut microbes associated with VFA and BMI might provide one explanation for why VFA is highly associated with metabolic risk factors [48] independent of BMI. Nonetheless, further research is needed to address how the results might explain the association between VFA and metabolic risk factors.

We observed that two microbial genera were significantly associated with VFA after adjusting for confounding factors (e.g., age, gender, smoking amount, alcohol consumption, dietary fiber intake, and medicine use). Changes in VFA were significantly and inversely associated with changes in the abundance ratios of *Blautia* and *Flavonifractor* (Table 2). However, changes in BMI were significantly and inversely associated with changes in the abundance ratios of *Alistipes*, *Clostridium XlVb*, *Erysipelotrichaceae incertae sedis*, and *Lactobacillus* (Table 2). These observations suggest that different gut microbial genera might be involved in VFA and BMI status. Furthermore, we identified effective gut microbial species in subjects with different types of obesity. This observation may provide a focus for future clinical trials, although further study is required for use in clinical trials. In our previous cross-sectional study [21], we found that *Blautia* is the only gut microbial genus that is significantly and negatively associated with VFA status, regardless of gender [21]. Therefore, one of the questions of the current longitudinal study was whether an increased abundance ratio of *Blautia* is associated with visceral fat reduction; indeed, we were able to confirm this. As for BMI, *Clostridium XlVb*, *Erysipelotrichaceae incertae sedis*, and *Lactobacillus* were significantly and negatively associated with BMI in both genders. We also identified *Alistipes*, a gut microbial genus that was not identified in the cross-sectional study, as being associated with changes in BMI. These findings suggest that cross-sectional and longitudinal studies are important to identify the role of gut microbes in obesity.

Among the gut microbial genera associated with a reduction in VFA, we identified two species that were significantly and negatively associated with VFA accumulation: *Blautia hansenii* and *Blautia producta* (Table 3). *Blautia* produces acetic acid and butyric acid [49], which are known to decrease obesity [50,51]. In addition, regardless of race, *Blautia* is one of the most abundant genera in the intestine [21,46,52,53]. However, *Blautia* is reportedly less prominent in obese children [54], diabetic adults, and pediatric patients [55,56], as well as subjects with other diseases, such as rectal cancer and rheumatoid arthritis [57,58], than in healthy subjects. The function of *Blautia hansenii* and *Blautia producta*, as compared with other *Blautia* species, are not well known; however, *Blautia producta* has been reported to be one of the important intestinal microflora species producing short-chain fatty acids [59]. Hence, although further study is needed to know whether changes in the abundance ratio of *Blautia hansenii* and *Blautia producta* in the gut affect the risk of metabolic disease, these species might be able to maintain or improve metabolic disease status, providing a new target and/or index against diabetes and obesity. Inulin [60] and resveratrol [61] are known to increase the abundance ratio of *Blautia*; therefore, high intakes of these foods may be one way to reduce visceral fat. Future mouse studies could augment the findings of this study. This time, *Flavonifractor* was significantly and negatively associated with changes in VFA using 16S rRNA sequences. However, *Flavonifractor plautii* was not associated with significant changes in VFA using metagenomic shotgun sequences. There are many species in the *Flavonifractor* genus [62]. Other *Flavonifractor* species may be associated with changes in VFA, so future studies are warranted.

One limitation of this study is that the data were collected via only two check-ups over a one-year interval. Even within one individual there is a lot of variation in the stool microbiome from day to day and spatially within each sample [63,64]; therefore, it might be necessary to confirm the reproducibility through an increase in the frequency of data collection and a longer study interval. We did not acquire crude negative controls of intestinal microflora [65]. Furthermore, as the current study was limited to one race and a single country, the reproducibility of the findings should be confirmed in a different race and/or country. Short chain fatty acids (SCFA) determination and gut permeability data were not obtained; however, they would be helpful to understand the observed associations.

## 5. Conclusions

The intestinal microflora composition is significantly associated with VFA or BMI; however, the associated gut microbes differ. Furthermore, two gut species—*Blautia hansenii* and *Blautia producta*—were significantly and negatively associated with VFA accumulation.

## Figures and Tables

**Table 1:** Baseline characteristics between the low-VFA group and high-VFA group.

Characteristics	Low-VFA			High-VFA			*p* Values *^a^
	Means		SDs	Means		SDs	
Visceral fat area (cm^2^)	50.7	±	17.1	116.9	±	31.0	
Age (y) *^b^	53.0	±	14.4	57.2	±	13.6	<0.001 **
Number (% female) *^c^	387 (77.8%)			380 (40.8%)			<0.001 **
Body mass index (kg/m^2^) *^b^	20.7	±	2.2	24.9	±	3.1	<0.001 **
Waist circumference (cm) *^b^	70.5	±	6.0	84.8	±	7.1	<0.001 **
Serum glucose (mg/dL) *^b^	4.3	±	0.6	4.8	±	1.0	<0.001 **
HbA1c (%) *^b^	5.6	±	0.3	5.9	±	0.7	<0.001 **
SBP (mmHg) *^b^	116.9	±	16.7	127.5	±	16.0	0.001 **
DBP (mmHg) *^b^	71.6	±	11.2	78.2	±	10.7	<0.001 **
Triglyceride (mg/dL) *^b^	0.9	±	0.4	1.4	±	1.0	<0.001 **
LDL cholesterol (mg/dL) *^b^	3.0	±	0.8	3.2	±	0.7	<0.001 **
HDL cholesterol (mg/dL) *^b^	1.9	±	0.4	1.6	±	0.4	<0.001 **
Smoking amount (stick/d) *^b^	4.7	±	10.9	7.9	±	11.1	<0.001 **
Amount of exercise (Mets/d) *^b^	4.0	±	10.6	6.6	±	15.8	0.038 *
Habitual medicine use (%Yes) *^c^	23.8%			42.3%			<0.001 **
Total energy intake (kcal/d) *^b^	1752.0	±	594.7	1858.0	±	667.5	0.002 **
Alcohol consumption (g/d) *^b^	7.8	±	16.1	14.0	±	20.0	<0.001 **
Total dietary fiber intake (g/d) *^b^	10.8	±	4.6	10.8	±	4.8	0.730

Data are presented as mean ± SD. *^a^ *p* < 0.05 and *p* < 0.01 are represented by * and **, respectively. *^b^ A Mann–Whitney U-test was used. *^c^ Test for equality of proportions was used.

**Table 2:** Association between the changes in intestinal microflora genus and changes in VFA or BMI assessed by 16S rRNA sequences.

	Changes in VFA ^a,b^			Changes in BMI ^a,c^		
Genus	β	(s.e.)	*p* Values ^d^	β	(s.e.)	*p* Values ^d^
*Alistipes*	−23.4	25.9	0.365	−4.2	2.0	0.038 *
*Blautia*	−36.2	14.9	0.015 *	−0.5	1.2	0.656
*Clostridium. XlVb*	−173.7	167.4	0.300	−30.7	13.0	0.019 *
*Erysipelotrichaceae incertae sedis*	−16.2	89.2	0.856	−46.2	6.6	<0.001 **
*Flavonifractor*	−486.1	200.3	0.016 *	−18.4	15.6	0.239
*Lactobacillus*	8.6	29.2	0.769	−5.6	2.3	0.014 *

^a^ Multiple regression analysis was used with changes in VFA/BMI as an objective variable; ^b^ For VFA, the following confounding factors were used for the adjustment: age, gender, VFA, and the abundance ratio of each intestinal microflora genus at the baseline; ^c^ For BMI, the following confounding factors were used for the adjustment: age, gender, BMI, and the abundance ratio of each intestinal microflora genus at the baseline; ^d^ *p* < 0.05 and *p* < 0.01 are represented by * and **, respectively. β was regression coefficient.

**Table 3:** Association between the changes in gut microbial species and changes in VFA assessed by metagenomic shotgun sequences.

	Changes in VFA ^a^		
Species	β	(s.e.)	*p* Values ^b^
*Blautia*			
*Blautia producta*	−26.16	7.70	<0.001 **
*Blautia hansenii*	−8.31	2.60	0.001 **
*Blautia* sp. *N6H1.15*	−10.35	8.01	0.197
*Blautia* sp. *SC05B48*	0.33	0.60	0.584
*Blautia* sp. *YL58*	−24.05	21.14	0.256
*Flavonifractor*			
*Flavonifractor plautii*	−1.28	0.85	0.131

^a^ Multiple regression analysis was used with changes in VFA/BMI as an objective variable. The following confounding factors were used for the adjustment: age, gender, VFA, the abundance ratio of each gut microbial species at baseline, changes in alcohol consumption, total fiber intake, smoking amount, exercise amount, BMI, and medicine use. ^b^ *p* < 0.01 are represented by **. β was the regression coefficient.

## Data Availability

The datasets generated and analyzed in the current study are available from the corresponding author on reasonable request.

## Supplementary Materials

The following are available online at https://www.mdpi.com/article/10.3390/biology11020318/s1, Figure S1: Changes in intestinal microflora composition over one year; Table S1: Association between changes in intestinal microflora composition and changes in VFA and BMI; Table S2. Association between changes in intestinal microflora genera and changes in VFA and BMI; Table S3. Adjusted association between changes in intestinal microflora l genera and changes in VFA and BMI; Table S4. Multiple regression analysis of the association between change in VFA and changes in *Blautia* or *Flavonifractor*, including related factors.

## References

[1] Remely M., Aumueller E., Merold C., Dworzak S., Hippe B., Zanner J., Pointner A., Brath H., Haslberger A.G. (2014). Effects of short chain fatty acid producing bacteria on epigenetic regulation of FFAR3 in type 2 diabetes and obesity. Gene.

[2] Hildebrandt M.A., Hoffmann C., Sherrill-Mix S.A., Keilbaugh S.A., Hamady M., Chen Y.Y., Knight R., Ahima R.S., Bushman F., Wu G.D. (2009). High-fat diet determines the composition of the murine gut microbiome independently of obesity. Gastroenterology.

[3] Turnbaugh P.J., Ley R.E., Mahowald M.A., Magrini V., Mardis E.R., Gordon J.I. (2006). An obesity-associated gut microbiome with increased capacity for energy harvest. Nature.

[4] Dore J., Simren M., Buttle L., Guarner F. (2013). Hot topics in gut microbiota. United Eur. Gastroenterol. J..

[5] Evans J.M., Morris L.S., Marchesi J.R. (2013). The gut microbiome: The role of a virtual organ in the endocrinology of the host. J. Endocrinol..

[6] David L.A., Maurice C.F., Carmody R.N., Gootenberg D.B., Button J.E., Wolfe B.E., Ling A.V., Devlin A.S., Varma Y., Fischbach M.A. (2014). Diet rapidly and reproducibly alters the human gut microbiome. Nature.

[7] Muegge B.D., Kuczynski J., Knights D., Clemente J.C., González A., Fontana L., Henrissat B., Knight R., Gordon J.I. (2011). Diet drives convergence in gut microbiome functions across mammalian phylogeny and within humans. Science.

[8] Wu G.D., Chen J., Hoffmann C., Bittinger K., Chen Y.-Y., Keilbaugh S.A., Bewtra M., Knights D., Walters W.A., Knight R. (2011). Linking long-term dietary patterns with gut microbial enterotypes. Science.

[9] Odamaki T., Kato K., Sugahara H., Hashikura N., Takahashi S., Xiao J., Abe F., Osawa R. (2016). Age-related changes in gut microbiota composition from newborn to centenarian: A cross-sectional study. BMC Microbiol..

[10] Mitsuoka T. (1992). Intestinal flora and aging. Nutr. Rev..

[11] Odamaki T., Kato K., Sugahara H., Hashikura N., Takahashi S., Xiao J., Abe F., Osawa R. (2012). Structure, function and diversity of the healthy human microbiome. Nature.

[12] Davenport E.R., Cusanovich D.A., Michelini K., Barreiro L.B., Ober C., Gilad Y. (2015). Genome-Wide Association Studies of the Human Gut Microbiota. PLoS ONE.

[13] Vijay-Kumar M., Aitken J.D., Carvalho F.A., Cullender T.C., Mwangi S., Srinivasan S., Sitaraman S.V., Knight R., Ley R.E., Gewirtz A.T. (2010). Metabolic syndrome and altered gut microbiota in mice lacking Toll-like receptor 5. Science.

[14] Caricilli A.M., Picardi P.K., de Abreu L.L., Ueno M., Prada P.O., Ropelle E.R., Hirabara S.M., Castoldi Â., Vieira P., Camara Niels O.S. (2011). Gut microbiota is a key modulator of insulin resistance in TLR 2 knockout mice. PLoS Biol..

[15] Ley R.E., Turnbaugh P.J., Klein S., Gordon J.I. (2006). Microbial ecology: Human gut microbes associated with obesity. Nature.

[16] Santacruz A., Collado M.C., García-Valdés L., Segura M.T., Martín-Lagos J.A., Anjos T., Martí-Romero M., Lopez R.M., Florido J., Campoy C. (2010). Gut microbiota composition is associated with body weight, weight gain and biochemical parameters in pregnant women. Br. J. Nutr..

[17] Schwiertz A., Taras D., Schäfer K., Beijer S., Bos N.A., Donus C., Hardt P.D. (2010). Microbiota and SCFA in Lean and Overweight Healthy Subjects. Obesity.

[18] Le Chatelier E., Nielsen T., Qin J., Prifti E., Hildebrand F., Falony G., Almeida M., Arumugam M., Batto J.-M., Kennedy S. (2013). Richness of human gut microbiome correlates with metabolic markers. Nature.

[19] Duncan S.H., Lobley G.E., Holtrop G., Ince J., Johnstone A.M., Louis P., Flint H.J. (2008). Human colonic microbiota associated with diet, obesity and weight loss. Int. J. Obes..

[20] Walker A.W., Ince J., Duncan S.H., Webster L.M., Holtrop G., Ze X., Brown D., Stares M.D., Scott P., Bergerat A. (2011). Dominant and diet-responsive groups of bacteria within the human colonic microbiota. ISME J..

[21] Ozato N., Saito S., Yamaguchi T., Katashima M., Tokuda I., Sawada K., Katsuragi Y., Kakuta M., Imoto S., Ihara K. (2019). Blautia genus associated with visceral fat accumulation in adults 20–76 years of age. NPJ Biofilms Microbiomes.

[22] Tilg H., Kaser A. (2011). Gut microbiome, obesity, and metabolic dysfunction. J. Clin. Investig..

[23] Arora T., Backhed F. (2016). The gut microbiota and metabolic disease: Current understanding and future perspectives. J. Intern. Med..

[24] Menni C., Jackson M.A., Pallister T., Steves C.J., Spector T.D., Valdes A.M. (2017). Gut microbiome diversity and high-fibre intake are related to lower long-term weight gain. Int. J. Obes..

[25] Tchernof A., Despres J.P. (2013). Pathophysiology of human visceral obesity: An update. Physiol. Rev..

[26] Karlsson T., Rask-Andersen M., Pan G., Höglund J., Wadelius C., Ek W.E., Johansson Å. (2019). Contribution of genetics to visceral adiposity and its relation to cardiovascular and metabolic disease. Nat. Med..

[27] Kuk J.L., Katzmarzyk P.T., Nichaman M.Z., Church T.S., Blair S.N., Ross R. (2006). Visceral fat is an independent predictor of all-cause mortality in men. Obesity.

[28] McNeely M.J., Shofer J.B., Leonetti D.L., Fujimoto W.Y., Boyko E.J. (2012). Associations among visceral fat, all-cause mortality, and obesity-related mortality in Japanese Americans. Diabetes Care.

[29] Koster A., Murphy R.A., Eiriksdottir G., Aspelund T., Sigurdsson S., Lang T.F., Gudnason V., Launer L.J., Harris T.B. (2015). Fat distribution and mortality: The AGES-Reykjavik study. Obesity.

[30] Matsushita Y., Nakagawa T., Yamamoto S., Takahashi Y., Yokoyama T., Noda M., Mizoue T. (2010). Associations of Visceral and Subcutaneous Fat Areas With the Prevalence of Metabolic Risk Factor Clustering in 6292 Japanese Individuals: The Hitachi Health Study. Diabetes Care.

[31] Shah R.V., Murthy V.L., Abbasi S.A., Blankstein R., Kwong R.Y., Goldfine A.B., Jerosch-Herold M., Lima J.A.C., Ding J., Allison M.A. (2014). Visceral Adiposity and the Risk of Metabolic Syndrome Across Body Mass Index. JACC Cardiovasc. Imaging.

[32] Ryo M., Maeda K., Onda T., Katashima M., Okumiya A., Nishida M., Yamaguchi T., Funahashi T., Matsuzawa Y., Nakamura T. (2005). A new simple method for the measurement of visceral fat accumulation by bioelectrical impedance. Diabetes Care.

[33] Daimon M., Kamba A., Murakami H., Mizushiri S., Osonoi S., Matsuki K., Sato E., Tanabe J., Takayasu S., Matsuhashi Y. (2017). Dominance of the hypothalamus-pituitary-adrenal axis over the renin-angiotensin-aldosterone system is a risk factor for decreased insulin secretion. Sci. Rep..

[34] Iino C., Shimoyama T., Chinda D., Sakuraba H., Fukuda S., Nakaji S. (2018). Infection of Helicobacter pylori and Atrophic Gastritis Influence Lactobacillus in Gut Microbiota in a Japanese Population. Front. Immunol..

[35] Iino C., Shimoyama T., Iino K., Yokoyama Y., Chinda D., Sakuraba H., Fukuda S., Nakaji S. (2019). Daidzein Intake Is Associated with Equol Producing Status through an Increase in the Intestinal Bacteria Responsible for Equol Production. Nutrients.

[36] Kumagai G., Wada K., Kudo H., Asari T., Chiba D., Ota S., Takeda O., Koyama K., Nakaji S., Ishibashi Y. (2019). Associations between cervical disc degeneration and muscle strength in a cross-sectional population-based study. PLoS ONE.

[37] Ozato N., Saito S., Yamaguchi T., Katashima M., Tokuda I., Sawada K., Katsuragi Y., Imoto S., Ihara K., Nakaji S. (2019). Association between Nutrients and Visceral Fat in Healthy Japanese Adults: A 2-Year Longitudinal Study Brief Title: Micronutrients Associated with Visceral Fat Accumulation. Nutrients.

[38] Ozato N., Saito S., Yamaguchi T., Katashima M., Tokuda I., Sawada K., Katsuragi Y., Kakuta M., Imoto S., Ihara K. (2020). Association between breath methane concentration and visceral fat area: A population-based cross-sectional study. J. Breath Res..

[39] Takahashi S., Tomita J., Nishioka K., Hisada T., Nishijima M. (2014). Development of a prokaryotic universal primer for simultaneous analysis of Bacteria and Archaea using next-generation sequencing. PLoS ONE.

[40] Sasaki S., Yanagibori R., Amano K. (1998). Self-administered diet history questionnaire developed for health education: A relative validation of the test-version by comparison with 3-day diet record in women. J. Epidemiol..

[41] Kobayashi S., Murakami K., Sasaki S., Okubo H., Hirota N., Notsu A., Fukui M., Date C. (2011). Comparison of relative validity of food group intakes estimated by comprehensive and brief-type self-administered diet history questionnaires against 16 d dietary records in Japanese adults. Public Health Nutr..

[42] Walkenhorst M.S., Reyes L., Perez G., Progulske-Fox A., Brown M.B., Phillips P.L. (2020). A Uniquely Altered Oral Microbiome Composition Was Observed in Pregnant Rats with Porphyromonas gingivalis Induced Periodontal Disease. Front. Cell. Infect. Microbiol..

[43] Bostanci N., Krog M.C., Hugerth L.W., Bashir Z., Fransson E., Boulund F., Belibasakis G.N., Wannerberger K., Engstrand L., Nielsen H.S. (2021). Dysbiosis of the Human Oral Microbiome During the Menstrual Cycle and Vulnerability to the External Exposures of Smoking and Dietary Sugar. Front. Cell. Infect. Microbiol..

[44] Japan Society for the Study of Obesity (2002). The Examination Committee of Criteria for ‘Obesity Disease’ in Japan. New criteria for ‘obesity disease’ in Japan. Circ. J..

[45] Brooks A.W., Priya S., Blekhman R., Bordenstein S.R. (2018). Gut microbiota diversity across ethnicities in the United States. PLoS Biol..

[46] Nishijima S., Suda W., Oshima K., Kim S.W., Hirose Y., Morita H., Hattori M. (2016). The gut microbiome of healthy Japanese and its microbial and functional uniqueness. DNA Res..

[47] Tanaka S., Horimai C., Katsukawa F. (2003). Ethnic differences in abdominal visceral fat accumulation between Japanese, African-Americans, and Caucasians: A meta-analysis. Acta Diabetol..

[48] Okauchi Y., Nishizawa H., Funahashi T., Ogawa T., Noguchi M., Ryo M., Kihara S., Iwahashi H., Yamagata K., Nakamura T. (2007). Reduction of visceral fat is associated with decrease in the number of metabolic risk factors in Japanese men. Diabetes Care.

[49] Liu C., Li J., Zhang Y., Philip A., Shi E., Chi X., Meng J. (2015). Influence of glucose fermentation on CO(2) assimilation to acetate in homoacetogen Blautia coccoides GA-1. J. Ind. Microbiol. Biotechnol..

[50] Kimura I., Inoue D., Maeda T., Hara T., Ichimura A., Miyauchi S., Kobayashi M., Hirasawa A., Tsujimoto G. (2011). Short-chain fatty acids and ketones directly regulate sympathetic nervous system via G protein-coupled receptor 41 (GPR41). Proc. Natl. Acad. Sci. USA.

[51] Kimura I., Ozawa K., Inoue D., Imamura T., Kimura K., Maeda T., Terasawa K., Kashihara D., Hirano K., Tani T. (2013). The gut microbiota suppresses insulin-mediated fat accumulation via the short-chain fatty acid receptor GPR43. Nat. Commun..

[52] Bamberger C., Rossmeier A., Lechner K., Wu L., Waldmann E., Fischer S., Stark R.G., Altenhofer J., Henze K., Parhofer K.G. (2018). A Walnut-Enriched Diet Affects Gut Microbiome in Healthy Caucasian Subjects: A Randomized, Controlled Trial. Nutrients.

[53] Zhang W., Li J., Lu S., Han N., Miao J., Zhang T., Qiang Y., Kong Y., Wang H., Gao T. (2019). Gut microbiota community characteristics and disease-related microorganism pattern in a population of healthy Chinese people. Sci. Rep..

[54] Benitez-Paez A., Gomez Del Pugar E.M., Lopez-Almela I., Moya-Perez A., Codoner-Franch P., Sanz Y. (2020). Depletion of Blautia Species in the Microbiota of Obese Children Relates to Intestinal Inflammation and Metabolic Phenotype Worsening. mSystems.

[55] Larsen N., Vogensen F.K., van den Berg F.W., Nielsen D.S., Andreasen A.S., Pedersen B.K., Al-Soud W.A., Sørensen S.J., Hansen L.H., Jakobsen M. (2010). Gut microbiota in human adults with type 2 diabetes differs from non-diabetic adults. PLoS ONE.

[56] Murri M., Leiva I., Gomez-Zumaquero J.M., Castellano-Castillo D., Moreno-Indias I., Urda-Cardona A., Tinahones F.J., Fernández-García J.C., Queipo-Ortuño M.I. (2013). Gut microbiota in children with type 1 diabetes differs from that in healthy children: A case-control study. BMC Med..

[57] Kakiyama G., Pandak W.M., Gillevet P.M., Hylemon P.B., Heuman D.M., Daita K., Takei H., Muto A., Nittono H., Ridlon J.M. (2013). Modulation of the fecal bile acid profile by gut microbiota in cirrhosis. J. Hepatol..

[58] Ohigashi S., Sudo K., Kobayashi D., Takahashi O., Takahashi T., Asahara T., Nomoto K., Onodera H. (2013). Changes of the intestinal microbiota, short chain fatty acids, and fecal pH in patients with colorectal cancer. Dig. Dis. Sci..

[59] Becker N., Kunath J., Loh G., Blaut M. (2011). Human intestinal microbiota: Characterization of a simplified and stable gnotobiotic rat model. Gut Microbes.

[60] Neyrinck A.M., Pachikian B., Taminiau B., Daube G., Frédérick R., Cani P.D., Bindels L.B., Delzenne N.M. (2016). Intestinal Sucrase as a Novel Target Contributing to the Regulation of Glycemia by Prebiotics. PLoS ONE.

[61] Wang P., Gao J., Ke W., Wang J., Li D., Liu R., Jia Y., Wang X., Chen X., Chen F. (2020). Resveratrol reduces obesity in high-fat diet-fed mice via modulating the composition and metabolic function of the gut microbiota. Free Radic. Biol. Med..

[62] Rodriguez-Castaño G.P., Rey F.E., Caro-Quintero A., Acosta-González A. (2020). Gut-derived *Flavonifractor* species variants are differentially enriched during in vitro incubation with quercetin. PLoS ONE.

[63] Ji B.W., Sheth R.U., Dixit P.D., Huang Y., Kaufman A., Wang H.H., Vitkup D. (2019). Quantifying spatiotemporal variability and noise in absolute microbiota abundances using replicate sampling. Nat. Methods.

[64] Vandeputte D., De Commer L., Tito R.Y., Kathagen G., Sabino J., Vermeire S., Faust K., Raes J. (2021). Temporal variability in quantitative human gut microbiome profiles and implications for clinical research. Nat. Commun..

[65] Salter S.J., Cox M.J., Turek E.M., Calus S.T., Cookson W.O., Moffatt M.F., Turner P., Parkhill J., Loman N.J., Walker A.W. (2014). Reagent and laboratory contamination can critically impact sequence-based microbiome analyses. BMC Biol..

