# Supplementary material for: Two Blautia Species Associated with Visceral Fat Accumulation: A One-Year Longitudinal Study
Source: Biology (Basel). 2022 Feb 16;11(2):318. doi: 10.3390/biology11020318 (PMC8869763; doi:10.3390/biology11020318)
Supplement: Supplementary file 1 [file biology-11-00318-s001.zip › biology-1584720-supplementary.pdf]

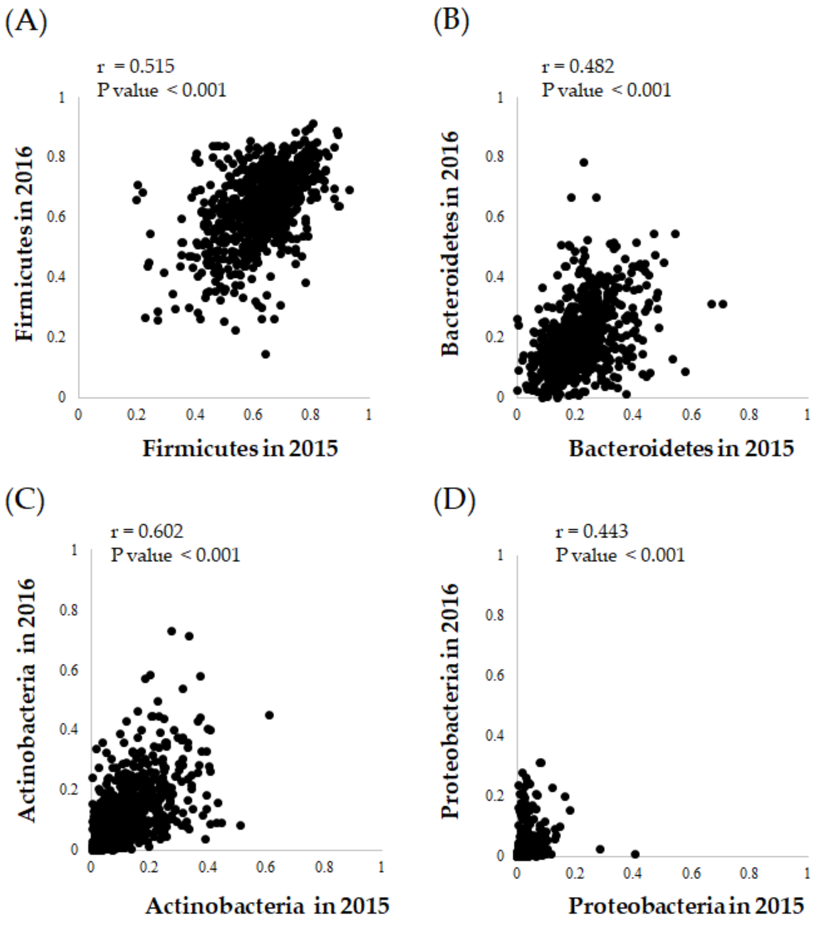

Figure S1. Changes in intestinal microflora composition in one year (N = 767). Four phyla, Firmicutes (**A**), Bacteroidetes (**B**), Actinobacteria (**C**), and Proteobacteria (**D**), account for the majority (~98%) of the human gut phyla. Spearman correlation was used to evaluate the association.

Table S1. Association between changes in intestinal microflora composition and changes in VFA and BMI.

Components	Explained variance [%]	Changes in VFA *P* for trend		Changes in BMI *P* for trend	
PC1	13.5	0.026*	(I)	0.293	(I)
PC2	11.8	0.390	(D)	0.446	(D)
PC3	9.4	0.471	(I)	0.175	(I)
PC4	8.2	0.189	(I)	0.357	(D)
PC5	7.5	0.075	(D)	0.009**	(D)

P for trend was evaluated by Jonckheere test. (D) indicates a tendency to decrease, while (I) indicates a tendency to increase in relation to VFA.

P < 0.05 and <0.01 are indicated by * and **, respectively.

Table S2. Association between changes in intestinal microflora genera and changes in VFA and BMI.

	Changes in VFA		Changes in BMI	
**Genus**	**R**	***P*-values**	**R**	***P*-values**
Alistipes	-0.060	0.098	-0.059	0.102
Anaerostipes	-0.013	0.717	0.040	0.265
Bacteroides	-0.009	0.807	-0.003	0.925
Bifidobacterium	0.085	0.018*	0.049	0.176
Blautia	-0.047	0.198	-0.035	0.327
Butyricicoccus	-0.021	0.563	-0.032	0.370
Clostridium.IV	0.011	0.756	-0.002	0.946
Clostridium.sensu.stricto	-0.004	0.902	0.017	0.629
Clostridium.XlVa	0.000	0.990	-0.055	0.125
Clostridium.XlVb	-0.037	0.303	0.018	0.609
Clostridium.XVIII	0.013	0.712	-0.017	0.631
Collinsella	0.020	0.575	-0.025	0.490
Dialister	0.043	0.232	0.044	0.228
Dorea	0.036	0.314	0.063	0.081
Eggerthella	-0.019	0.601	-0.071	0.049*
Erysipelotrichaceae incertae sedis	-0.037	0.311	-0.030	0.401
Escherichia/Shigella	0.034	0.352	-0.006	0.859
Faecalibacterium	0.010	0.777	0.038	0.292
Flavonifractor	-0.098	0.007**	-0.077	0.033*
Fusicatenibacter	0.009	0.805	-0.002	0.964
Gemmiger	0.003	0.941	-0.045	0.209
Intestinibacter	0.001	0.985	0.023	0.527

Lachnospiracea incertae sedis	0.040	0.264	-0.016	0.665
Lactobacillus	0.026	0.474	-0.055	0.127
Odoribacter	-0.038	0.293	-0.037	0.302
Oscillibacter	-0.031	0.391	-0.072	0.045
Parabacteroides	-0.025	0.496	-0.008	0.820
Phascolarctobacterium	0.001	0.969	0.026	0.474
Prevotella	-0.031	0.391	-0.046	0.206
Romboutsia	0.058	0.108	0.036	0.321
Roseburia	0.010	0.787	0.051	0.158
Ruminococcus	-0.001	0.978	-0.048	0.188
Streptococcus	0.025	0.481	0.028	0.435
Sutterella	-0.009	0.811	0.027	0.454
Turicibacter	-0.010	0.775	-0.019	0.603
Veillonella	0.025	0.485	0.089	0.014

Spearman was used.

P < 0.05 and <0.01 are indicated by * and **, respectively.

Table S3. Adjusted association between changes in intestinal microflora l genera and changes in VFA and BMI.

	Changes in VFA		Changes in BMI	
**Genus**	**β**	***P*-values**	**β**	***P*-values**
Alistipes	–23.449	0.365	–4.171	0.038*
Anaerostipes	11.659	0.328	1.460	0.115
Bacteroides	–5.155	0.590	0.513	0.491
Bifidobacterium	11.402	0.153	0.053	0.932
Blautia	–36.189	0.015*	–0.520	0.656
Butyricicoccus	–128.407	0.295	–54.272	0.695
Clostridium.IV	18.660	0.281	–0.848	0.530
Clostridium.sensu.stricto	–78.195	0.261	–1.481	0.784
Clostridium.XlVa	–96.466	0.550	–16.368	0.193
Clostridium.XlVb	–173.652	0.300	–30.693	0.019*
Clostridium.XVIII	–63.564	0.359	–5.464	0.312
Collinsella	–6.200	0.686	1.004	0.401
Dialister	174.615	0.103	2.966	0.722
Dorea	79.906	0.406	12.565	0.092
Eggerthella	–201.284	0.444	–24.598	0.229
Erysipelotrichaceae incertae sedis	–16.244	0.856	–46.209	<0.001**
Escherichia/Shigella	38.438	0.272	3.550	0.188
Faecalibacterium	6.276	0.591	1.171	0.196
Flavonifractor	–486.139	0.016*	–18.445	0.239
Fusicatenibacter	41.656	0.137	0.401	0.854
Gemmiger	4.839	0.860	0.427	0.841
Intestinibacter	–64.557	0.587	2.755	0.766
Lachnospiracea incertae sedis	37.402	0.393	0.655	0.848
Lactobacillus	8.578	0.769	–5.574	0.014*
Odoribacter	–336.166	0.294	–26.357	0.291
Oscillibacter	–46.310	0.628	–26.357	0.291
Parabacteroides	–83.617	0.188	–4.903	0.322
Phascolarctobacterium	10.539	0.897	5.042	0.424
Prevotella	–5.816	0.405	0.080	0.883
Romboutsia	63.004	0.374	5.004	0.365
Roseburia	10.841	0.421	1.719	0.101
Ruminococcus	–12.835	0.379	–0.397	0.727
Streptococcus	12.859	0.343	1.968	0.687
Sutterella	56.780	0.365	1.968	0.687
Turicibacter	–104.343	0.120	–0.024	0.996
Veillonella	12.526	0.760	2.871	0.368

Multiple regression analysis was used with changes in VFA/BMI as an objective variable.

As for VFA, the following factors were used for the adjustment: age, sex, VFA, and the abundance ratio of each gut microbiota genus at baseline.

As for BMI, the following factors were used for the adjustment: age, sex, BMI, and the abundance ratio of each gut microbiota genus at baseline.

$P < 0.05$ and <0.01 are indicated by * and **, respectively.

Table S4. Multiple regression analysis of the association between change in VFA and changes in *Blautia* or *Flavonifractor*, including related factors.

	**Changes in VFA**	
	**β**	***P*-values**
**For *Blautia***		
Change in the abundance ratios of *Blautia*	-34.66	0.017*
Change in alcohol consumption	-0.04	0.311
Change in total fiber intake	0.08	0.576
Change in smoking habits	0.03	0.725
Change in BMI	3.36	<0.001**
Change in medicine use	-0.82	0.742
Abundance ratios of *Blautia* at the baseline	25.61	0.107
Age at the baseline	0.00	0.953
Sex at the baseline	-5.50	<0.001**
VFA at the baseline	-0.07	<0.001**
**For *Flavonifractor***		
Change in the abundance ratios of *Flavonifractor*	-435.40	0.026*
Change in alcohol consumption	-0.05	0.272
Change in total fiber intake	0.09	0.506
Change in smoking habits	0.05	0.610
Change in BMI	3.29	<0.001**
Change in medicine use	-0.46	0.852
Abundance ratios of *Flavonifractor* at the baseline	-164.70	0.421
Age at the baseline	0.01	0.813
Sex at the baseline	-5.91	<0.001**
VFA at the baseline	-0.07	<0.001**

β = regression coefficient

P < 0.05 and <0.01 are indicated by * and **, respectively.
